# Altered Metabolic and Stemness Capacity of Adipose Tissue-Derived Stem Cells from Obese Mouse and Human

**DOI:** 10.1371/journal.pone.0123397

**Published:** 2015-04-13

**Authors:** Laura M. Pérez, Aurora Bernal, Beatriz de Lucas, Nuria San Martin, Annalaura Mastrangelo, Antonia García, Coral Barbas, Beatriz G. Gálvez

**Affiliations:** 1 Centro Nacional de Investigaciones Cardiovasculares (CNIC), Madrid, Spain; 2 Universidad San Pablo CEU, Madrid, Spain; 3 Universidad Europea de Madrid (UEM), Madrid, Spain; University of California, San Diego, UNITED STATES

## Abstract

Adipose stem cells (ASCs) are an appealing source of cells for therapeutic intervention; however, the environment from which ASCs are isolated may impact their usefulness. Using a range of functional assays, we have evaluated whether ASCs isolated from an obese environment are comparable to cells from non-obese adipose tissue. Results showed that ASCs isolated from obese tissue have a reduced proliferative ability and a loss of viability together with changes in telomerase activity and DNA telomere length, suggesting a decreased self-renewal capacity. Metabolic analysis demonstrated that mitochondrial content and function was impaired in obese-derived ASCs resulting in changes in favored oxidative substrates. These findings highlight the impact of obesity on adult stem properties. Hence, caution should be exercised when considering the source of ASCs for cellular therapies since their therapeutic potential may be impaired.

## Introduction

Adipose tissue has several distinct anatomical locations within the body and the differential expansion of these depots is of great importance to the study of obesity and insulin resistance. Adipose tissue plays an active role in metabolic homeostasis and functions as a central endocrine organ [1,2]. In addition to its occupation in energy storage *via* lipid buffering, adipose tissue releases various proteins that help control a number of metabolic pathways. Although chiefly composed of adipocytes, it is now acknowledged that adipose tissue is a significant reservoir of mesenchymal stem cells, termed adipose-derived stem cells (ASCs) [3].

ASCs are prominent tools in regenerative medicine, both for their multipotent capacity and their ease of isolation [4]. Accordingly, ASCs can differentiate into several tissue lineages, such as adipocytes, osteocytes and muscle cells, highlighting their utility in stem cell therapy. Indeed, several clinical trials have tested the ability of ASCs to treat different disorders, including myocardial infarction [5], cartilage or bone formation [6], and for fat grafting in plastic surgery [7].

Self-renewal is the process by which stem cells divide to create more stem cells [8]. It is clear that therapeutic applications of MSCs rely heavily on maintenance of the key stem cell properties, proliferation capacity and multilineage differentiation potential, during culture and expansion. These *stemness* attributes are essential for tissue homeostasis and pluripotency, such as protection from the acquisition of mutations that accumulate with every round of DNA replication [9,10]. Recent studies have shown that the core *stemness* factors, Nanog and Oct4, are associated with the undifferentiated pluripotent state of stem cell populations derived from various adult tissues [11]. Moreover, it has been reported that hypoxia inducible factor 1-α (HIF-1α), a hypoxia-triggered broad-range transcription factor, is similarly involved in regulating fundamental cellular processes, including stemness, proliferation and differentiation [12].

Autologous stem cell therapy represents a powerful option for regenerative cell-based treatment. Recent studies have considered the limitations in the therapeutic potential of ASCs by different processes such as diabetes and aging [13,14]. Indeed, we demonstrated previously that ASCs from an obese environment have impaired differentiation and migration properties [15,16]. However, many questions remain unanswered regarding the best source of therapeutic cells. To further explore the apparent inequalities of obese-derived ASCs, we have examined the metabolic and stemness properties of the ASC reservoir. Our results suggest that obesity leads to a general collapse in the homeostasis regulatory network of ASCs. This data support the caveat that while adipose tissue is a convenient source of ASCs, obesity has to be considered when using these cells for regenerative medicine applications.

## Research Design and Methods

### Reagents

Dulbeco’s modified Eagle’s medium (DMEM) was purchased from Sigma (St. Louis MO), supplemented with 10% FBS (Sigma). Penicillin, streptomycin, L-glutamine and Hepes was from Lonza (Basel, Switzerland). Antibodies to Nanog and *β*-actin were from Abcam (Cambridge, UK). Mitotracker Green and Mitosox Red were purchased from Molecular Probes, Invitrogen (Carlsbad, CA). TOPRO-3, DAPI (4’,6-diamino-2-phenylindole dihydrochloride) and ProLong Antifade reagent were from Invitrogen. The Violet Ratiometric Membrane Asymmetry Probe/Dead Cell Apoptosis Kit was from Invitrogen and EpiSeeker hydroxymethylated DNA Quantification Kit from Abcam. Kits for molecular studies were purchased from Applied Biosystems, Life Technologies (Paisley, UK). DIO rodent purified HFD (“Diet induced Obesity” with 60% energy from fat, formula 58Y1) was obtained from TestDiet (IPS Product Supplies Ltd, London, UK). Unless otherwise stated, all other reagents were purchased from Sigma-Aldrich.

### Isolation and culture of ASCs

ASCs were obtained from nonobese or obese murine and human subjects and characterized as described previously [15–17]. Briefly, ASCs were isolated from adipose tissue close to vessels by the “explant technique”; these ASCs are mesenchymal stem cells with a closely related endothelial origin (CD34+). Mouse subcutaneous adipose tissue was collected from adult C57BL6 female mice maintained on chow diet (nonobese ASCs), or from mice fed a high fat diet (HFD) for 4 months (obese ASCs) (Table 1). Mice were maintained and used in accordance with the National Institutes of Health Animal Care and Use committee and protocols with mice were approved by the National Center for Cardiovascular Research ethics committee.

**Table 1:** Body weight average in mice and BMI average in human groups.

Mean (±SD)	Nonobese	Obese
Mice	22.50 (2.10)	36.22 (4.30)
Human	20.00 (2.21)	34.00 (3.10)

n = 5 (values in grams for mice and in kg for human).

Human ASCs were obtained from nonobese patients (BMI < 22 kg/m^2^) and from obese patients (BMI > 30 kg/m^2^) (Table 1). Human subcutaneous adipose tissue was obtained from patients after bariatric surgery at the Hospital Universitario de la Princesa, Madrid (Females aged between 35 and 45 years), with a total of five nonobese patients (BMI < 22 kg/m^2^) and five obese (BMI > 30 kg/m^2^). Verbal informed consent was obtained from all subjects (Normal procedure in 2009 for verbal consent was approved by the local ethics committee) and the sample collection conformed to the principles set out in the WMA Declaration of Helsinki and the NIH Belmont Report. The use of human cell samples was approved by the Centro Nacional de Investigaciones Cardiovasculares ethics committee.

ASCs were isolated, sorted and expanded from mouse and human tissues as described [3]. Cells were cultured in DMEM supplemented with 10% FBS at 37°C in a humidified 5% CO_2_/95% air atmosphere. Cells at passage 10 or 20 were used for experiments as indicated.

### Cell proliferation assays

Cell growth was assessed manually by cell counting. Cells were seeded in triplicate into 24 multi-well plates at a density of 2.5x10^3^ cells/cm^2^ in DMEM plus 10% FBS, and were counted each day during 4 days. Cell morphology was analyzed by flow cytometry with an LSRFortessa cytometer (BD Biosciences, San Jose, CA) using forward-scatter (FSC) and side-scatter (SSC) profiles [18]. Cell proliferation was also quantified with the BdrU Incorporation Assay Kit. Briefly, 4000 cells were seeded in 96-multiwell plates and BrdU was added to each well for 16 h. Cells were then fixed and stained with an anti-BrdU antibody, followed by incubation with anti-mouse IgG HRP and peroxidase substrate. Incorporation of BrdU into the cell was measured with a Benchmark Plus microplate spectrophotometer (Bio Rad, Hercules CA, USA) at 450 nm and 540 nm.

### Cell viability assessment

Apoptosis was measured using the Violet Ratiometric Membrane Asymmetry Probe/Dead Cell Apoptosis Kit (Invitrogen) as described [19]. Both floating and adhered cells were collected and pelleted by centrifugation. Adhered cells were detached with trypsin. Cells were suspended in Hanks Balanced Salt Solution (HBSS) containing 4'-N,N-diethylamino-6-(N,N,N-dodecyl-methylamino-sulfopropyl)-methyl-3-hydroxyflavone (F2N12S, 200 nM) plus SYTOX AAdvanced dead cell stain solution (1 mM) at 10^6^ cells/ml and incubated for 5 min at room temperature in the dark. Apoptosis measurements were performed with an LSRFortessa cytometer (BD Biosciences, San Jose, CA) at 405 nm and 488 nm. Data were analyzed with BD FACSDiva Software.

### Analysis of mitochondrial content and reactive oxygen species

Mitochondrial content and reactive oxygen species (ROS) were measured as described [20]. Briefly, cells were washed in PBS, detached with trypsin and incubated at 10^6^ cells/ml in HBSS containing 100 nM Mitotracker Green or 1 μM Mitosox Red for 30 min in the dark. Viability was assessed by incubation with TOPRO-3. For each sample, at least 10000 cells were analyzed for the corresponding fluorescence in a BD LSRFortessa cytometer (BD Biosciences, San Jose, CA) and data were analyzed with FACSDiva Software.

Mitotracker Green was also used to visualize the mitochondrial network. Cells were plated on 0.1% gelatin-coated coverslips, washed with PBS and fixed with cold methanol for 10 min. Next, cells were stained with 100 nM Mitotracker Green for 1h at 37°C. Following incubation, cells were stained with 300 nM DAPI for 30 min and coverslips were mounted (ProLong) on glass slides. Images were obtained with a Leica DM2500/TCS SPE confocal microscope (Leica Microsystems, Wetzlar, Germany).

### Measurement of Respiration and Acidification Rates

Oxygen consumption rate (OCR) and extracellular acidification rate (ECAR) were measured in cells using a Seahorse XF-96 analyzer (Seahorse Bioscience, North Billerica, MA) as described [21]. ASCs were seeded in cell culture microplates at 5000 cells/well for mouse and 7000 cells/well for human in 100 μl growth medium, and then incubated at 37°C for 24 h. Assays were initiated by replacing growth medium with 175 μl of pre-warmed assay medium: a low-buffered RPMI medium containing 25 mM glucose and 1 mM pyruvate, to measure glycolytic activity, or containing 0.5 mM carnitine, 50 μM palmitate and 1 mM pyruvate, to measure β-oxidation activity. Cells were incubated at 37°C for 30 min to allow media temperature and pH to reach equilibrium. Following equilibration, OCR in pMoles/min (indicator of mitochondrial respiration) and ECAR in mpH/min (indicator of lactic acid production or glycolysis) were measured simultaneously for 2 min to establish a baseline rate. After baseline measurement, 25 μl of the following reagents prepared in assay medium were then injected into each well to give the desired final concentration: Port A 0.125 μM oligomycin, a mitochondrial respiration uncoupler; Port B 0.03 μM FCCP, a mitochondrial accelerator; and, Port C 0.8 μM rotenone and antimycin, complex I and III inhibitors, respectively). New OCR and ECAR values were then determined. Two baseline rates and two response rates were measured and the average of two baseline rates or test rates was used for data analysis. At the end of the assay, cells were detached with trypsin and the number and percentage of viable cells was determined using trypan blue. OCR and ECAR profiles define the measurements in this study (S1 Fig). *Basal respiration* is considered as the total cellular resting O_2_ consumption. *Maximal Respiratory Capacity* is the maximum amount of O_2_ that can be consumed through the respiratory chain. *Basal Acidification Rate* is the cellular lactate levels produced. *Maximum Acidification Rate* is the maximum rate of lactate produced from glycolysis when the mitochondrial ATP synthase is inhibited.

### Measurement of lactic acid in supernatants

A Lactate Assay Kit for lactic acid measurements was purchased from Sigma (St. Louis MO). Briefly, 5000 cells were seeded in 96-multiwell plates. Then, 10 μl of supernatant from each well of the cultured cell plate was transferred to a new plate, followed by incubation with 50 μl of reaction solution containing the substrate, cofactor and enzyme mixture. The amount of lactate release into the culture medium was measured with a Benchmark Plus microplate spectrophotometer (Bio Rad, Hercules CA, USA) at 570 nm. Data were normalized to total protein amount.

### Q-TRAP assay

Telomerase activity was measured as described [9]. Briefly, cellular protein was extracted in NP40 lysis buffer (10 mM Tris-HCl, 1 mM MgCl_2_, 1 mM EDTA, 1% NP40, 0.25 mM sodium deoxycholate, 10% glycerol, 150 mM NaCl, 5 mM *β*-mercaptoethanol) and 1 × protease inhibitor (Roche, Rotkreuz, Switzerland) at pH 8.0. Protein concentration was measured with the DC Protein Assay Kit (Bio-Rad, Hercules, CA). Extracts (5 and 1μg protein per sample) were mixed with telomerase extension buffer (500 mM Tris-AcH, 500 mM potassium acetate, 30 mM MgCl_2_, 10 mM spermine, 10 mM EGTA, 50 mM *β*-mercaptoethanol, 2 mM dAGT) and 1mM Oligo TS (5′-AATCCGTCGAGCAGAGTT-3′). A 2μl aliquot of this extension reaction was added to 23μl PCR reaction mix containing 1 × Power SYBR Green PCR Master Mix, 5 mM EGTA, 2 ng/μl Oligo TS and 4 ng/μl Oligo ACX (5′-GCGCGGC(TTACCC)4-3′). PCR was carried out at 94°C for 10 min, followed by 40 cycles of 94°C for 15 s and 60°C for 1 min. PCR products were monitored with an AB 7900 Fast Real-Time PCR System and quantified using SDS 2.0 software (Applied Biosystems).

### Telomere length quantification by Q-FISH

Telomere length was measured by qFISH using a Cy3-labeled LL(CCCTAA)3 PNA telomeric probe (Eurogentec, Liège, Belgium). Following hybridization, slides were washed three times with PBS-0.1% Tween for 10 min at 60°C and dehydrated through an ethanol series (70, 90 and 100%, 5 min each). Slides were then counterstained and mounted in Vectashield H-1200 mounting medium (Vector Laboratories, Burlingame, CA). Digital images were acquired with a Leica DM2500/TCS SPE confocal microscope and camera set-up. Telomere signals were captured with the same exposure time in all samples, from at least 20–30 nuclei per group, and were quantified using the TFL-Telo software (version 2) (Vancouver, Canada).

### Gene Expression Profiling

Total RNA was extracted from ASCs with TRI Reagent and reverse transcribed using the High Capacity cDNA Reverse Transcription Kit. Quantitative expression qPCR was performed using the following primer pairs: β-Actin (forward [Fw], CACGATGGAGGGGCCGGACTCAT; reverse [Rv], TAAAGACCTCTATGCCAACACAG), mTet1 (Fw, AGCTACCCTGAGTTTCACCC; Rv, CAATTAGGCGCTGTCTGTCC), hTet1 (Fw, CCAGTGAAAGAGGCATCTCC; Rv, TCTTCAGTGGAGCTGGTGTG), mTet2 (Fw, TAGCTTTGCGTCAGTGGAGA; Rv, AGGGATGGCTGGCTCAAAA), hTet2 (Fw, GCTGGAGCACAAGTCACAAA; Rv, GAGAAGTGCACCTGGTGTGA), mTet3 (Fw, TTCCCTACCTGCGATTGTGT; Rv, AGAACTCTTCCCCTCCTTGC), hTet3 (Fw, CCCAGAGCTCCAACTGCTAC; Rv, ACAGAGGTGTCTTGCCCATC), mNanog (Fw, CTTACAAGGGTCTGCTACTGAGATGC; Rv, TGCTTCCTGGCAAGGACCTT), hNanog (Fw, AATACCTCAGCCTCCAGCAGATG; Rv, CTGCGTCACACCATTGCTATTCT), mOct4 (Fw, TCTTCTGCTTCAGCAGCTTG; Rv, GTTGGAGAAGGTGGAACCAA), hOct4 (Fw, CGAAAGAGAAAGCGAACCAGTAT; Rv, AGCAGCCTCAAAACCCTCTCGTT), mSox2 (Fw, AAAGCGTTAATTTGGATGGG; Rv, ACAAGAGAATTGGGAGGGGT), hSox2 (Fw, GTCATTTGCTGTGGGTGATG; Rv, AGAAAAACGAGGGAAATGGG), HIF1α (Fw, CGTTCCTTCGATCAGTTGTC; Rv, TCAGTGGTGGCAGTGGTAGT), mp53 (Fw, TCCGACTGTGACTCCTCCAT; Rv, CTAGCATTCAGGCCCTCATC), hp53 (Fw, TGGCCATCTACAAGCAGTCA; Rv, GGTACAGTCAGAGCCAACCT), mp21 (Fw, CAAAGTGTGCCGTTGTCTCT; Rv, AAGTACTGGGCCTCTTGTCC), hp21 (Fw, CCCAAGCTCTACCTTCCCAC; Rv, CTGAGAGTCTCCAGGTCCAC). Each cDNA sample was amplified in triplicate using a SYBR Green PCR Master Mix. PCR mixes were loaded in an AB 7900 Fast Real-Time PCR System and quantified using SDS 2.0 software.

### Western blot analysis

Whole cell lysates were extracted with lysis buffer containing 50 mM Tris (pH 8), 150 mM NaCl, 1 mM EDTA, 0.1% Triton X-100 and 0.25% sodium deoxycholate and 1 × protease inhibitor cocktail (Roche, Rotkreuz, Switzerland). Protein samples were resolved by 10% SDS-PAGE and gels were transferred to nitrocellulose membranes (GE Healthcare, Little Chalfont, UK). Membranes were blocked with 5% nonfat milk for 1h at room temperature and subsequently incubated overnight at 4°C with 1:1000 primary antibodies to Nanog and β-actin. Signals were detected using the appropriate horseradish peroxidase-conjugated secondary antibody (Dako, Glostrup, Denmark). Blots were visualized by an enhanced chemiluminescence system (GE Healthcare, Little Chalfont, UK), and relative intensity of the specific bands was quantified by densitometry using NIH ImageJ software (http://rsb.info.nih.gov/ij/).

### DNA sample preparation

Genomic DNA was purified from 10^6^ cells with TRI Reagent followed by precipitation with ethanol. Purified genomic DNA was washed and suspended in TE buffer (10 mM Tris-HCL, 1 mM EDTA, pH 8.0).

### DNA methylation (5-methylcytosine) quantification

5-methylcytosine (5-hmC) was quantified using the EpiSeeker hydroxymethylated DNA Quantification Kit (Abcam) as described [22].

### Metabolite analysis

Equal numbers of each cell line were used to obtain extracts (five biological replicates). For each replicate, 10^6^ cells were collected by gentle scraping with a rubber cell scraper, suspended in cold methanol extraction solvent on dry ice and vortexed for 1 min. Samples were subjected to three freeze–thaw cycles for complete cell disruption, allowed to sit for 10 min at 4°C, placed in liquid nitrogen for 10 min and thawed in an ice bath for 10 min with brief vortexing. The supernatant was recovered by centrifugation at 5725 *g* for 5 min at 4°C and the pellet was re-extracted two times.

Preparation of cellular extracts and culture medium for LC-MS and GC-MS analysis was carried out as described previously [23], except that 100 μl sample volumes were used for GC-MS analysis. Samples were analyzed by GC-EI-Q-MS and by UHPLC-ESI-MS-QTOF (Agilent Technologies, Waldbronn, Germany) and data were examined as reported previously for GC-EI-Q-MS [23] and UHPLC-ESI-MS-QTOF [24]. After an appropriate statistical analysis, only the variables statistically significant (*p*-value <0.05) were identified putatively. For GC-MS analysis, metabolites were identified by comparing retention time, retention index and mass fragmentation patterns with those available in an in-house library comprehensive of both the NIST mass spectral library 2008 and the Fiehn RTL library (Agilent Technologies). For LC-MS data, the variables were identified by searching their accurate masses in the public databases: METLIN (www.metlin.scripps.edu), KEGG (www.genome.jp/kegg), LIPIDMAPS (www.lipidmaps.org/), which were all simultaneously accessed by an in-house-developed search engine, CEU MassMediator (http://ceumass.eps.uspceu.es/mediator).

Data quality for LC-MS and GC-MS was assured by using the quality control (QC) samples for GC-MS and LC-MS analysis as reported [24]. The QCs (n = 10 for each analytical technique) were prepared by pooling equal volumes of each sample. QCs were treated and injected along with the individual samples. Data processing was done together with the real samples as described above. As supportive information, the uniformity of some supplementary variables (pressure curve of the analyses within the same batch and the IS) was assured.

### Data analysis

Statistical analysis was performed using the GraphPad Prism software package (GraphPad, San Diego, CA.). Comparison of groups was performed with two-way analysis of variance. Values were expressed as mean±SEM or mean±SD, and data was considered significantly different when p < 0.05. Concerning data from GC-MS and LC-MS, the statistical analysis was performed by applying both univariate and multivariate data analysis using Matlab software (Mathworks, Natick, MA, USA) and SIMCA P+ 12.0.1 software (Umetrics, Umea, Sweden), respectively. For the univariate data analysis (UVDA), the non-parametric Mann−Whitney U test was applied to all the comparisons and the variables with a *p*-value lower than 0.05 were selected as significantly changed.

## Results

### Obese conditions modify ASC viability

**Fig 1:**
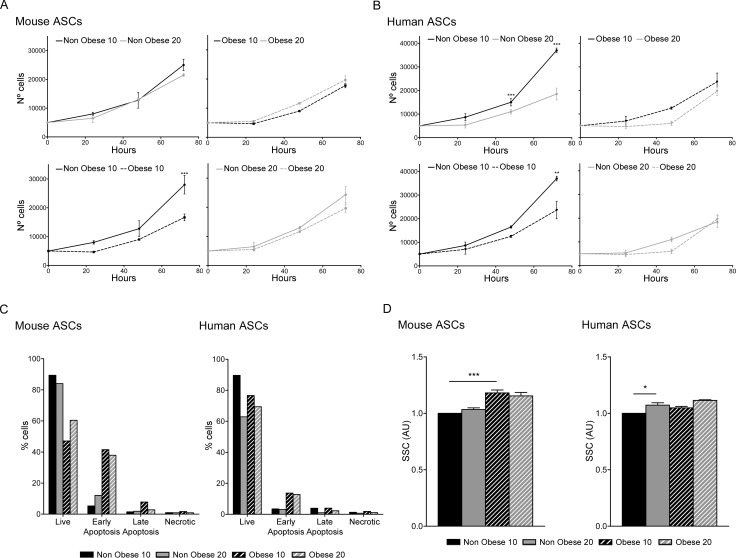
Proliferation and viability of ASCs. Proliferation was measured in murine (A) and human (B), at passage 10 and 20 nonobese and obese ASCs by cell counting. Data represent mean±SEM from three replicates. Cell death was measured using a radiometric assay kit and quantified by flow cytometry (C). One representative experiment of three independent experiments is shown. Morphological analysis by flow cytometry showing cell size (SSC) (D). Data represent mean±SEM from three independent experiments. * P < 0.05; ** P < 0.01; *** P < 0.001. AU, arbitrary units.

Our previous results established that ASCs from an obese environment are compromised in their ability to differentiate and migrate [15,16]. To assess whether they were also restricted in their capacity to proliferate, murine and human ASCs were isolated from both obese and nonobese adipose tissue as described [15–17], and their proliferative capacity was measured during early and late culture passage. At early passage (passage 10), both murine and human nonobese ASCs displayed faster growth kinetics than those from obese environments (Fig 1A and 1B). The population doubling time (PDT), calculated from growth curves, was greater in mouse obese ASCs (34.23 h at p10 and 32.81 h at p20) than in nonobese (30.1 h at p10 and 31.95 h at p20) and also greater in human obese ASCs (29.91 h at p10 and 31.57 h at p20) compared with nonobese ASCs (23.6 h at p10 and 34.17 h at p20). In addition, growth rates obtained from PDT values (S2A Fig) were comparable with results obtained from BrdU incorporation (S2B Fig). In contrast, the culture passage did not affect the proliferation rate of nonobese, obese mouse and obese human ASCs, but significantly affected human nonobese ASCs, with a clear reduction in proliferation at extended passage (Fig 1B).

To determine whether the differences in growth rates were related to changes in cell viability during proliferation, we measured apoptosis in cells using a kit to detect membrane asymmetry changes. In both murine and human cultures, the percentage of early apoptotic cells was higher in obese ASCs relative to nonobese ASCs (at both early and late passage), and the percentage of live cells decreased to approximately 40% in both obese murine and human cell cultures (Fig 1C).

To evaluate the possible obesity-induced morphological changes in ASCs associated with adipose tissue expansion, we analyzed cell size and complexity by flow cytometry. Results showed that both mouse and human obese ASCs displayed increased cell size and structural complexity compared with nonobese cells (Fig 1D). Collectively, these results show that ASCs taken from an obese environment present a reduced proliferative capacity with hypertrophy, and an increase in apoptosis that reduces their viability. These alterations may be associated with a loss of cell homeostasis.

### Obesity alters mitochondrial mass and function of ASCs

**Fig 2:**
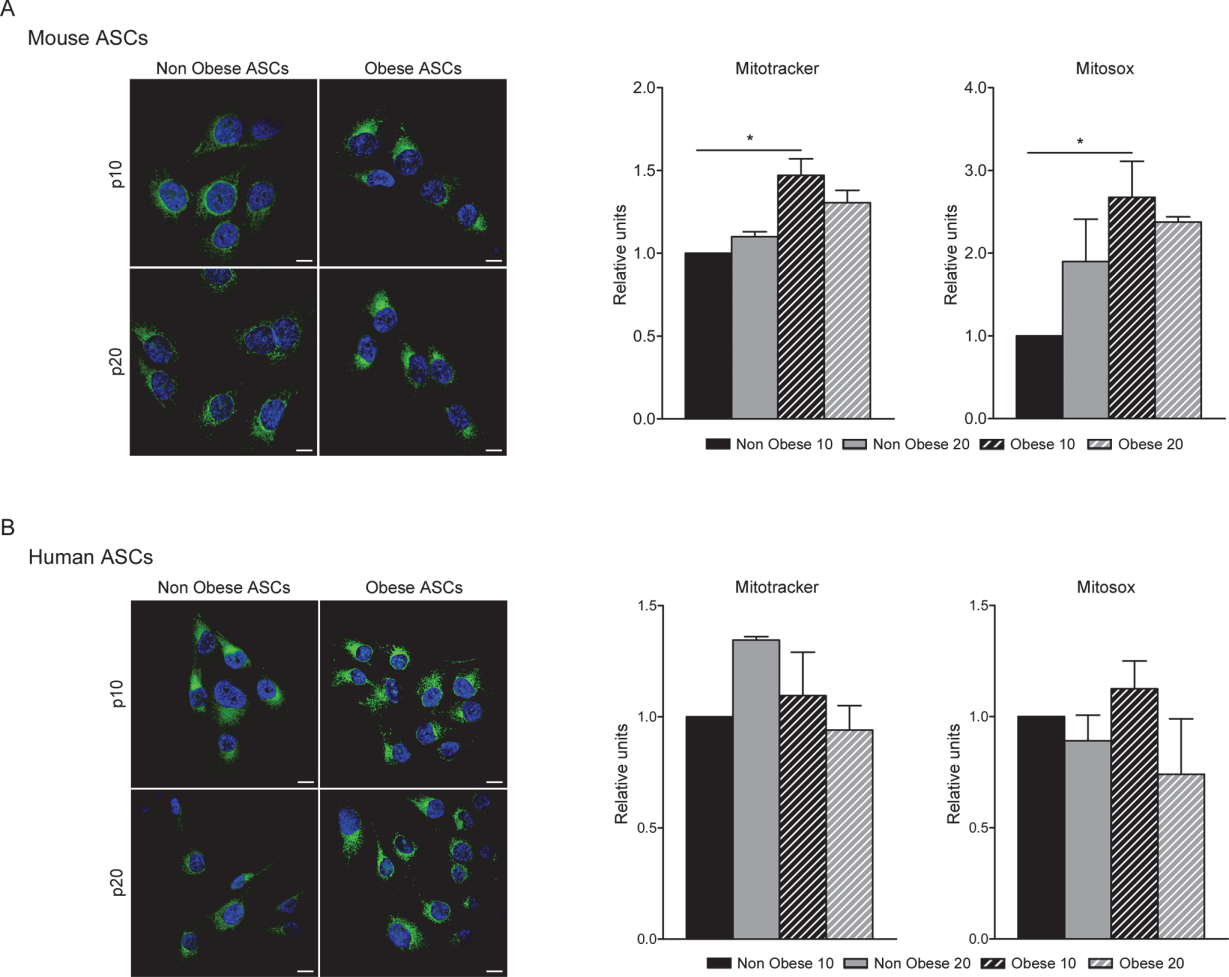
Mitochondrial abundance in ASCs. Confocal microscopy of Mitotracker Green staining in murine (A) and human (B) ASCs. One representative image out of three independent experiments is shown. Bar, 10 μm. Mitochondria quantification by flow cytometry with Mitotracker Green and ROS production with Mitosox Red in murine (A) and human ASCs (B). Data represent mean±SEM from three independent experiments. * P < 0.05.

Several studies have reported that obstruction of mitochondrial function may lead to changes in proliferation and the cell cycle [25,26]. Adequate mitochondrial performance is necessary to meet cellular demand and supports cell proliferation of stem cells [27]. Given the evident differences in ASC proliferation, we assessed mitochondrial function in cells isolated from nonobese and obese environments. Using Mitotracker Green to stain mitochondria, we first assessed mitochondrial mass in nonobese and obese ASCs at both early and late passage. By confocal microscopy, we observed increased Mitotracker Green staining in early passage murine and human obese ASCs (Fig 2A and 2B), which was particularly evident in obese human ASCs both at early and late passage (Fig 2B). Moreover, flow cytometry analysis demonstrated a significant increase in the abundance of mitochondria in murine obese ASCs (Fig 2A), whereas mitochondrial abundance in human ASCs was unchanged under obese conditions (Fig 2B). Next, we analyzed the mitochondrial redox status using the ROS-specific Mitosox Red dye. Consistent with the observed increase in mitochondrial mass in murine ASCs, early passage obese ASCs presented significantly higher levels of ROS compared to nonobese ASCs (Fig 2B), whereas obesity did not alter ROS levels in human cells.

**Fig 3:**
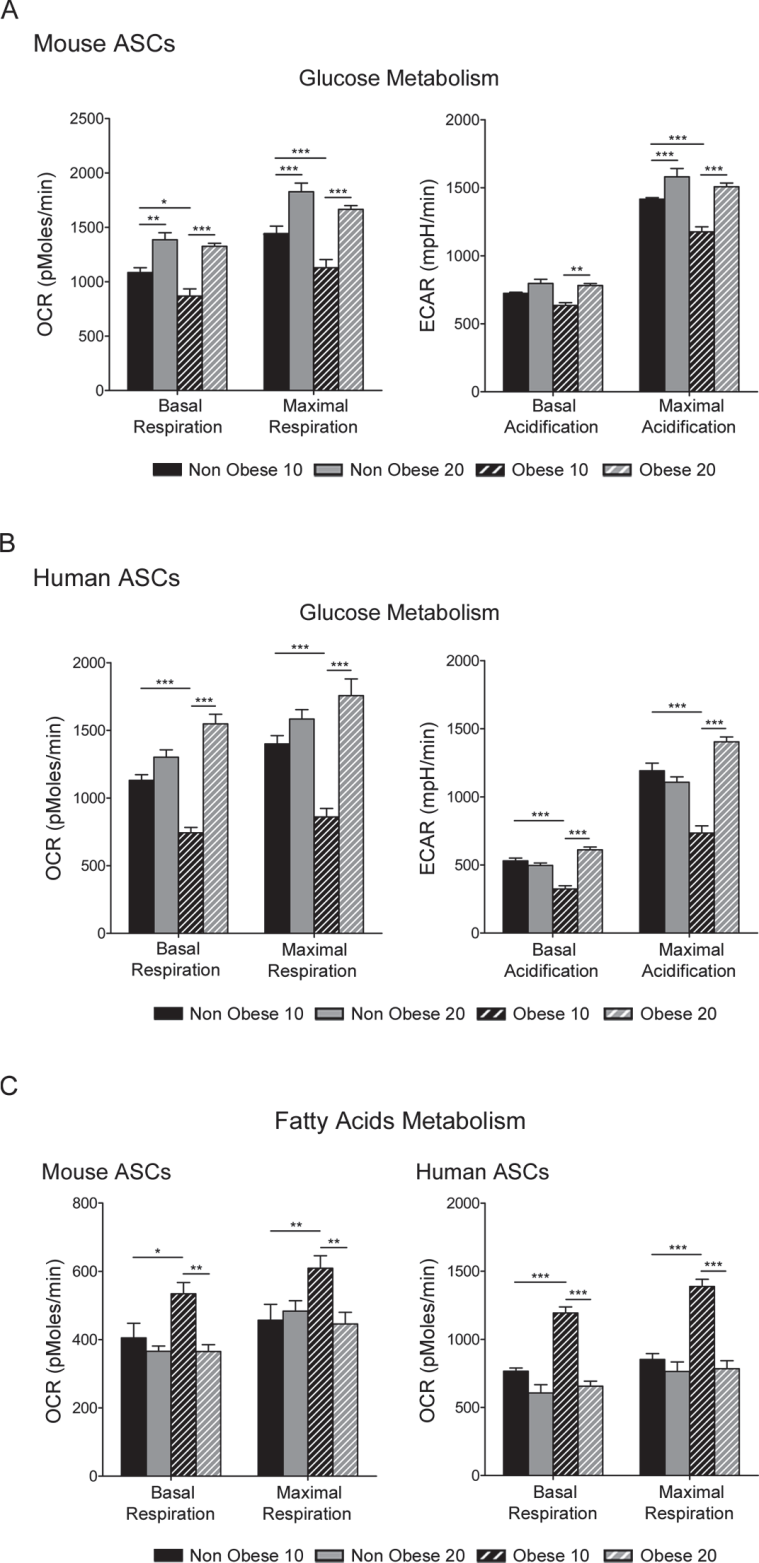
Glucose and free fatty acid metabolism in ASCs. Mitochondrial respiration analysis in murine (A and C) and human (B and C) ASCs. Oxygen consumption rate (OCR) and acidification levels (ECAR) during glucose metabolism (A and B) or fatty acid metabolism (C) were measured. Data represent mean±SEM from one representative experiment with six replicates. * P < 0.05; ** P < 0.01; *** P < 0.001.

To evaluate the consequences of the dissimilar mitochondrial load in obese ASCs, we measured cellular respiration as a metric of mitochondrial function. Thus, oxygen consumption rate (OCR), an indicator of respiration, and extracellular acidification rate (ECAR), a proxy for glycolysis, was measured using the Seahorse Bioscience XF96 instrument. Additionally, we challenged ASCs with alternative carbon fuels to assess glucose and free fatty acid metabolism. When challenged with glucose, both OCR and ECAR were greater in nonobese murine ASCs compared with obese ASCs (Fig 3A), and comparable results were found with human ASCs (Fig 3B). Interestingly, when glucose was replaced with fatty acids as the carbon source, both obese murine and human ASCs exhibited higher OCR values relative to nonobese ASCs (Fig 3C). When early and late passage cells were compared, late nonobese murine ASCs showed increased OCR and ECAR values relative to early nonobese murine ASCs with glucose as a carbon source, whereas OCR and ECAR values were similar in human ASCs under these conditions. In contrast, using fatty acids as a carbon source, no differences were observed in OCR values in late passage nonobese cells. In late obese ASCs, a similar response to early nonobese cells (both murine and human) was found (Fig 3C). Thus, ASCs from an obese environment displayed altered glucose metabolism. Measurement of cellular lactate concentration revealed that lactate production by murine and human obese ASC was reduced compared with nonobese ASCs (S3A Fig). These results correlated with the decrease in glucose respiration observed in obese ASCs. In contrast, the increased ECAR values obtained in the respiration measurements revealed an extracellular acidification that could be contributed by respiratory CO_2_ and by monocarboxylates other than lactate [21,28,29].

Collectively, these results indicate that although obese ASCs contain a greater number of mitochondria and produce more ROS, the mitochondria are not being used efficiently for oxygen consumption. Forcing cells to use β-oxidation (rather than glycolysis) for energy production revealed that obese ASCs could utilize this carbon source more capably than nonobese ASCs.

### Obesity leads to DNA damage and aging in ASCs

**Fig 4:**
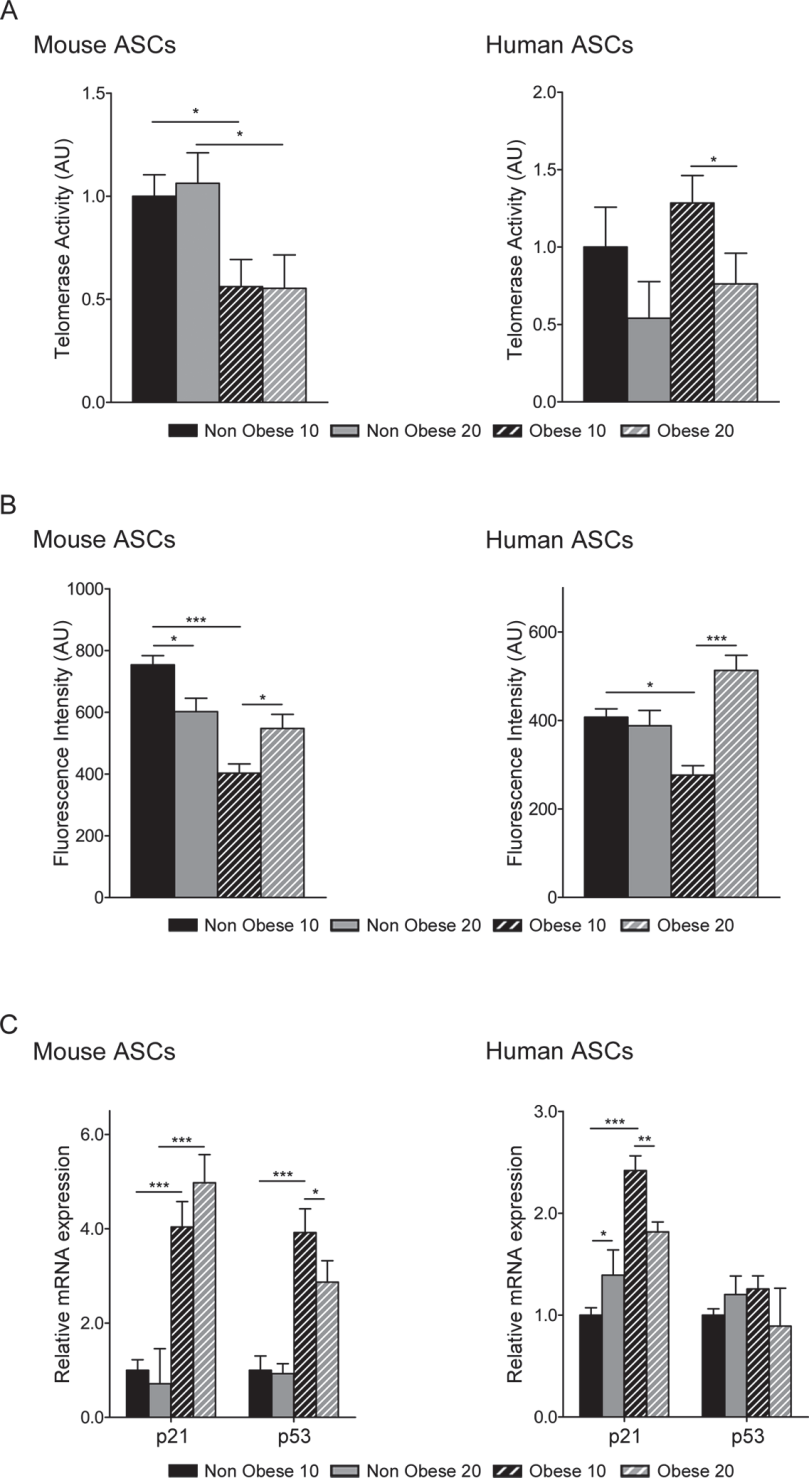
Comparison of age related parameters in ASCs from nonobese and obese environments. Relative telomerase activity in murine and human ASCs measured by qTRAP assay (A). Data represent mean±SEM from three independent experiments. Telomere length was measure by qFISH (B) in murine and human ASCs. Data represent mean fluorescence intensity ± SEM from 20 representative images. Gene expression profile of cell cycle proteins, p53 and p21 (C). Data represent mean±SEM from three independent experiments. * P < 0.05; ** P < 0.01; *** P < 0.001. AU, arbitrary units.

Oxidative damage is considered a primary determinant of aging. Thus, we questioned whether the increased mitochondria mass and ROS could accelerate the aging process in obese ASCs *in vitro*. We first evaluated telomerase activity by qTRAP assay. ASCs from murine obese adipose tissue exhibited a decrease in telomerase activity, at both short-term and long-term passages, compared with nonobese murine cells (Fig 4A). Surprisingly, human but not murine telomerase activity decreased with prolonged passage, which was significant for obese ASCs. The origin of these changes is not known but might perhaps be related to the low levels of this enzyme found in human mesenchymal stem cells [30,31]. Given the reduction of telomerase activity, we expected to discover a gradual shortening of telomeres in obese ASCs. As anticipated, we found that telomere length in both murine and human obese ASCs decreased at short-term passage, and telomere length decreased with passage in murine nonobese ASCs, but not in human nonobese ASCs (Fig 4B). Interestingly, both murine and human obese ASCs increased their telomere length with passage (Fig 4B).

DNA damage can arrest proliferation by activating apoptosis or senescence, preventing the transmission of DNA alterations and also diminishing self-renewal in stem cells. These biological events depend on an up-regulation of the cell-cycle inhibitor p21 and, as a consequence, the down-regulation of the tumor suppressor p53 [32]. To investigate p21 and p53 regulation in obese conditions, we evaluated their expression levels in ASCs. Expression of p21 was significantly increased in murine and human obese ASCs, and p53 levels were also increased in murine but not human obese ASCs (Fig 4C). These findings indicate that murine obese ASCs increased the mRNA levels of p21 and p53, and p21 was increased in human obese ASCs, presumably leading to apoptosis (Fig 1C). Collectively, these results suggest that an obese environment negatively affects DNA stability, which impacts on telomere length and apoptosis.

### Self-renewal and pluripotency of ASCs

**Fig 5:**
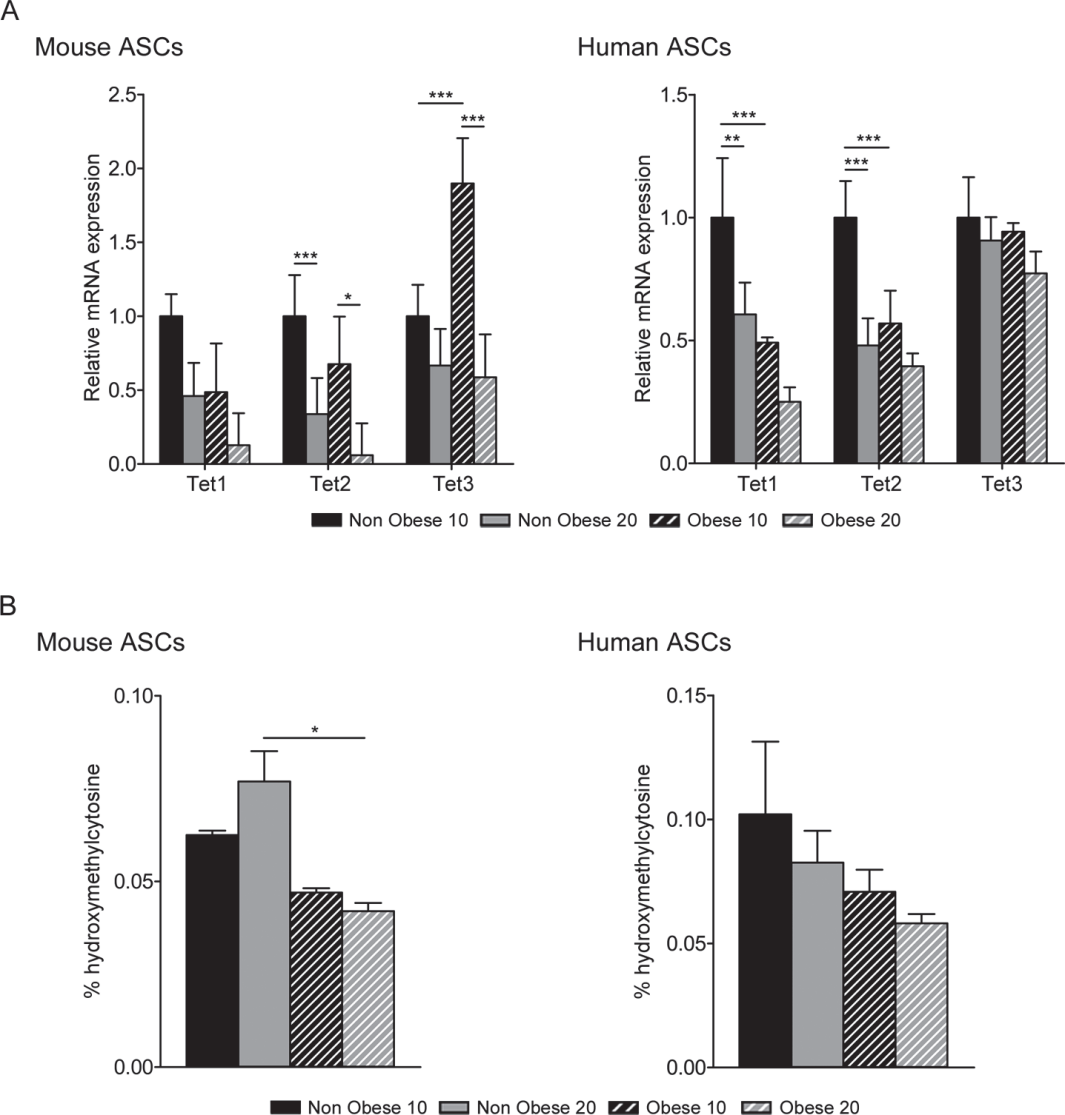
Hydroxymethylation in ASCs. Quantitative Real Time PCR was performed to evaluate TET family gene expression in murine and human ASCs (A). Data represent mean±SEM from three different experiments. Levels of 5-hydroxymethylcytosine were measured by EpiSeeker hydroxymethylated DNA Quantification Kit in murine and human ASCs (B). * P < 0.05; ** P < 0.01; *** P < 0.001.

TET family proteins are responsible for DNA demethylation through conversion of 5-methylcytosine (5mC) to 5-hydroxymethylcytosine (5hmC), and play an important role in stem cell self-renewal and maintenance [33]. To determine whether obesity caused alterations in the self-renewal capacity of ASCs, we measured the expression pattern of TET genes by qPCR. Compared with nonobese cells, we found a statistically-significant decrease in the level of TET1 and TET2 expression in human obese ASCs, whereas no significant differences were found in murine obese ASCs (Fig 5A). Moreover, no differences in TET3 expression were found in human obese ASCS, but there was a decrease of TET3 in murine late passage obese ASCs (Fig 5A).

Given that expression of TET results in the generation of 5hmC, we examined the correlation between the expression levels of TET proteins and 5hmC enrichment. To assess the enrichment of the 5hmC fraction of DNA, we employed a single-capture and detection antibody assay. As expected, the percentage of 5hmC decreased significantly in mouse obese ASCs at both passages, but not in human (Fig 5B). These results show that the decrease in TET1 protein in murine obese ASCs correlates with the reduction of global 5hmC levels in murine obese ASCs.

**Fig 6:**
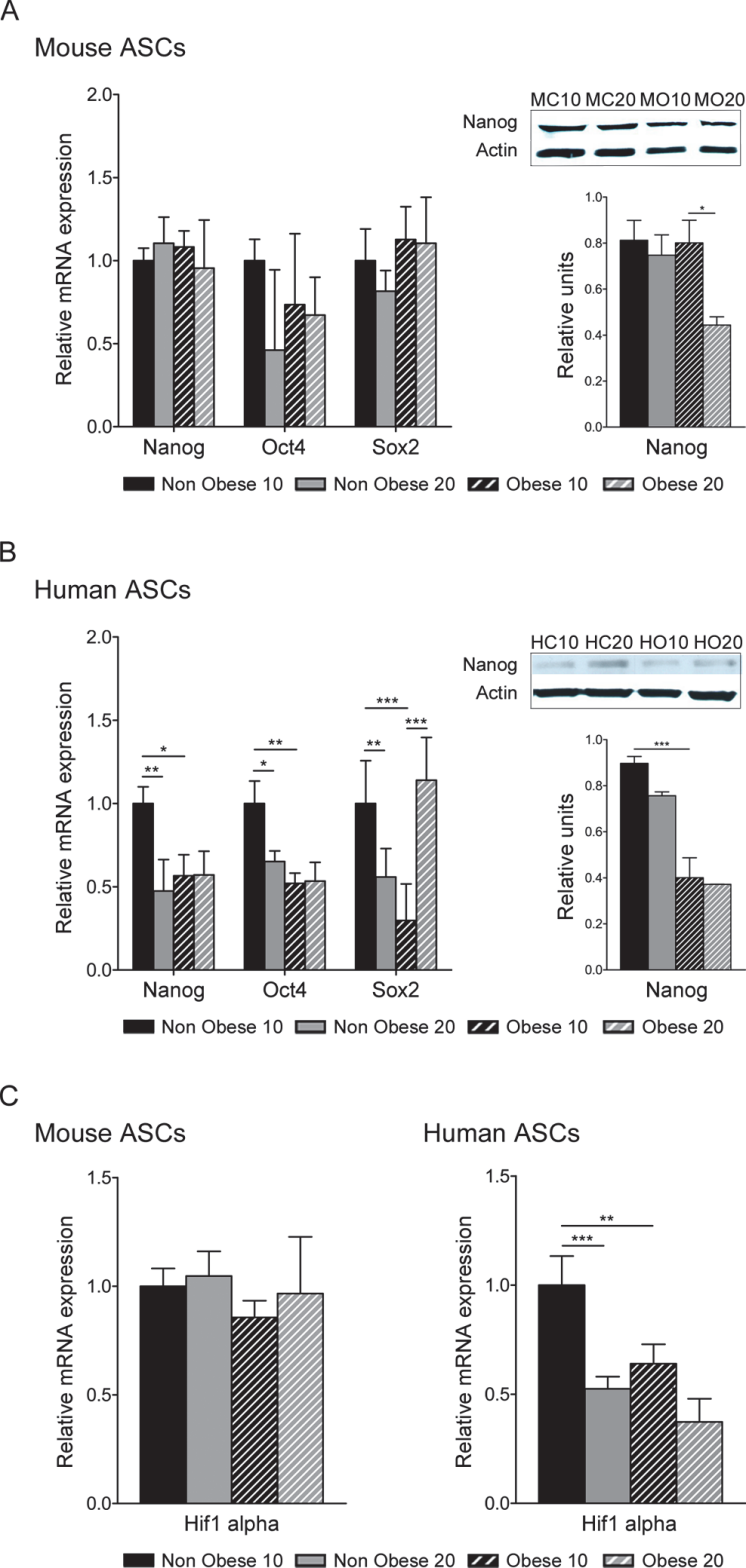
Expression analysis of related genes. Quantitative Real Time PCR was performed on mRNA extracted from nonobese and obese ASCs at passage 10 and 20, to evaluate expression of stem cell markers, Nanog, Oct4 and Sox2 (A, B) and Hif1α (C). Western blotting was performed to measure the relative protein expression of Nanog (A, B). Image representative and graph represent mean±SEM from four different experiments. * P < 0.05; ** P < 0.01; *** P < 0.001. MC10, mouse nonobese ASCs at passage 10; MC20, mouse nonobese ASCs at passage 20; MO10, mouse obese ASCs at passage 10; MO20, mouse obese ASCs at passage 20; HC10, human nonobese ASCs at passage 10; HC20, human nonobese ASCs at passage 20; HO10, human obese ASCs at passage 10; HO20, human obese ASCs at passage 20.

TET1 plays a central role in murine embryonic stem cell biology by maintaining Nanog expression [34]. Thus, we next measured expression of the key pluripotency genes, Nanog, Oct-4 and Sox-2, in ASCs. All three transcription factors were expressed in all cultured ASCs (Fig 6A and 6B). Whereas expression levels of the three factors were generally decreased in obese human ASCs, levels in murine obese ASCs were comparable to equivalent nonobese cells (Fig 6A and 6B). In accordance with the results obtained from mRNA expression, immunoblotting of total extracts revealed a marked decrease in Nanog protein abundance in human obese ASCs, and also a minor decrease in murine obese ASCs. (Fig 6A and 6B). These results suggest that obesity decreases the expression of pluripotency genes, correlating with a decrease in the hydroxymethylation quantity in DNA.

### Metabolic changes in obese ASCs

Having shown that obesity produces a switch in cellular energy metabolism, implying fundamental changes in bioenergetic pathways, we next surveyed the metabolite landscape in cells and in culture medium (Tables 2 and 3). Examination of intracellular metabolites revealed significant changes in the levels of several metabolites in both mouse and human obese ASCs, including a 35% reduction in leukotrienes (proinflammatory chemokines [35]) in murine obese ASCs, and a 65% reduction in human ASCs. Moreover, we found a 60% reduction of creatine, an antioxidant molecule [36], in murine obese ASCs and a 29% reduction in human obese ASCs. This result supports the finding of impaired cytokine production and redox imbalance in obese ASCs, leading to metabolic dysfunction. Interestingly, analysis of extracellular metabolites revealed an increase in glucose and glutamic acid in both in murine and human obese ASCs, supporting the finding of impaired glucose uptake in obese ASCs [15]. Moreover, results revealed a decrease in mysistic acid in both murine and human obese ASCs (Table 3), supporting the increase in fatty acid uptake and corroborating the changes observed in oxygen consumption in response to fatty acid respiration.

**Table 2:** Compounds significantly altered in obese versus nonobese ASCs.

	Name	RT (min)	Experimental mass	Mass error (ppm)	*p*-value	Change Ob vs Nob [%]	RSD for Qcs[%]	Abundance* Ob	Abundance* Nob
Mouse	Leukotriene-A4	4.11	340.2016	0	2.51E-03	-35	6	5.8E+05	8.9E+05
	Creatine	0.27	131.0696	0	8.51E-05	-60	2	1.7E+06	4.2E+06
Human	Leukotriene-A4	4.11	340.2016	0	6.05E-03	-65	6	1.4E+05	4.0E+05
	Creatine	0.27	131.0696	0	2.09E-02	-24	2	1.4E+06	1.9E+06

*Total Abundance

**Table 3:** Compounds significantly altered in culture medium of obese versus nonobese ASCs.

	Name	RT (min)	Experimental mass	Mass error (ppm)	*p*-value	Change Ob vs Nob [%]	RSD for Qcs[%]	Abundance Ob	Abundance Nob
Mouse	myristic acid	21.97	343	-	-	-100	21	ND	4.8E-02
	fatty acyl glycoside	8.11	604.4841	12	1.58E-02	31	6	1.7E+05	1.3E+05
	2-monostearin	24.95	399	-	-	-100	13	ND	7.8E+00
	fructose/glucose†	0.24	202.0455	0	1.07E-03	30	4	8.7E+06	6.7E+06
	glutamic acid†	0.41	129.0428	1	8.16E-03	31	15	1.5E+07	1.1E+07
	phenilalanine†	0.26	165.079	0	1.58E-02	38	9	1.7E+06	1.2E+06
Human	myristic acid	21.97	343	-	3.69E-02	-42	21	8.1E-02	1.4E-01
	fatty acyl glycoside	8.11	604.4841	12	1.58E-02	100	6	2.5E+05	1.3E+05
	2-monostearin	24.95	399	-	3.69E-02	-41	13	6.2E+00	1.0E+01
	fructose¥	17.08	103	-	3.69E-02	89	19	1.5E-06	7.4E-07
	glutamic acid¥	13.15	156	-	2.30E-02	141	20	2.92E+01	1.21E+01
	phenilalanine¥	14.43	218	-	1.58E-02	68	21	4.69E+00	2.80E+00

ND: not detected

†:LC-MS analysis

¥:GC-MS analysis

Obese conditions are thought to be associated with adipose tissue hypoxia, which can trigger insulin resistance in adipocytes [37]. To question the presence of Hif1α, a key mediator of the hypoxia response, expression levels were determined in ASCs in normoxic culture. Hif1α mRNA expression was significantly reduced in human obese ASCs compared with non-obese cells, and also in cells at higher passage, but no differences were detected in murine ASCs (Fig 6C).

Together, this analysis suggests that obese ASCs show a decrease in glucose uptake, a reduced pro-inflammatory and antioxidant protection, and altered normoxic levels of HIF1α.

## Discussion

Here we demonstrate that an obese environment modulates the *in vitro* properties of ASCs. We have previously shown that ASCs isolated from obese subjects have significantly reduced capacities for differentiation, migration and angiogenic potential [15,16]. These results are consistent with the differences in growth rates and adipogenic potential contingent on the anatomical distribution of the adipose tissue [38], and a decrease in the adipose tissue reservoir of active stem cells in obese patients [39].

ASCs derived from an obese environment present alterations in their proliferative ability, displaying a lower *in vitro* proliferation rate [40]. The increase in cell population doubling time and changes observed in their morphology (increase in cell size and structural complexity) are associated with increased apoptosis (Fig 1). Our findings indicate that ASCs derived from obese adipose tissue show a loss of cell homeostasis. Our results are bolstered by the recent finding that obese conditions can reduce the adipogenic potential of ASCs, as well as their proliferative ability [18]. Although ASCs from nonobese and obese subjects show similar mesenchymal surface markers, major alterations can be observed in metabolic pathways. Interestingly, we found that ASCs from obese mouse and human adipose tissue contained a greater quantity of mitochondria and produce more ROS (Fig 2). It is acknowledged that mitochondrial function in adipocytes is essential for their biology, and mitochondrial-generated ROS can be linked to an alteration in insulin sensitivity [41]. Additionally, high levels of ROS are accompanied by increased adiposity and lead to apoptosis [42]. In our study, the increased quantities of mitochondria and ROS were inversely proportional to proliferation and directly proportional to apoptosis [43]. Moreover, although mitochondrial load was greater in obese ASCs, oxygen consumption was reduced, at least under normal conditions in glucose-supplemented media. In contrast, oxygen consumption was higher in obese ASCs when free fatty acids were used as a carbon source in the respiration process (Fig 3). Thus, obese conditions altered mitochondrial biogenesis, increased ROS production and increased the extracellular acidification, contributing to the mitochondrial dysfunction effects associated with insulin resistance. We have previously shown that ASCs have an impaired ability to store lipids and produce adipokines [15]. Thus, a generalized dysfunctional metabolic phenotype is characteristic of obese ASCs.

In vitro culture of obese ASCs leads to alterations in mitochondrial mass and function. Under obese conditions, mitochondrial mass and ROS production is increased and there is a decrease in oxygen consumption in glycolysis and an increase in oxygen consumption in β-oxidation. In contrast, ASCs isolated from nonobese tissue present lower mitochondrial mass and ROS production but oxygen consumption is increased compared with obese ASCs in glycolysis, but not in β-oxidation. An alteration in mitochondrial biogenesis may be impaired by age-dependent accumulation of point mutations [44] that might alter oxidative phosphorylation and mitochondrial density in obese and nonobese ASCs in singular ways.

Having established the effect of obesity in adipose stem cell metabolism, we studied its possible relationship with aging [45,46]. Obesity is linked to oxidative stress and inflammation, and the cellular environment impacts on telomere length [47]. We observed a decrease in telomere length in mouse obese ASCs, but no significant differences were found in human ASCs (Fig 4). Interestingly, an obese environment affected telomerase activity in mouse ASCs. This *in vitro* finding likely reflects an inactivation of telomerase activity that leads to a decrease in telomere length. In the human population, there are many psychological stresses that may systemically affect cell aging [48]. These factors should therefore be taken into account when measuring telomere length in humans.

It has been reported that some cell cycle-related proteins, such as p53/p21, p16INK4A/Rb, and Pten/p27Kip1, are involved in cellular senescence [49,50]. Although we observed an effect in proliferation and apoptosis, we were unable to detect senescence with the histochemical marker β-galactosidase. However, we found that obesity increased p53 and p21 proteins, presumably leading to apoptosis. In contrast, p16 levels did not increase, suggesting that obese conditions result in apoptosis but not senescence in ASCs.

Environmental exposure can alter the expression of genes *via* epigenetic modifications [51]. Accordingly, DNA modifications by methylation and 5-hydroxymethylation can regulate gene expression in embryonic stem cells [33]. Stem cells maintain their self-renewal capacity in culture, but under obese conditions they might lose their stemness properties. We demonstrate an association between obesity, epigenetic marks and stemness. Obese ASCs presented a reduction in 5hmC levels together with a decrease in the expression levels of TET1 and TET2 enzymes (Fig 5) [34,52]. Moreover, alterations in the hydroxymethylation pattern coincided with changes in pluripotency gene expression. Accordingly, we observed a decrease in the mRNA expression levels of Nanog, Oct4 and Sox2 in obese ASCs (Fig 6), and also a significant reduction in the level of Nanog protein. TET genes are key players in pluripotency gene regulation [53,54] and TET gene expression correlates with passage in ASCs; consequently, we observed a decrease in TET expression and hydroxymethylation levels together with a decrease in pluripotency markers.

Inflammation associated-obesity has been shown to change the microenvironment and trigger chronic alterations in different adipose stem cell capacities. It is known that HIF plays key roles in this development and affects cell survival, cell-cycle and metabolism [55]. *Hif1α* expression was reduced in obese ASCs and also after passage in human obese ASCs (Fig 6C) but none in murine obese ASCs. These results might indicate a relationship between the observed deleterious functions of obese ASCs and HIF1α. In normal culture (normoxia), obese ASCs have impaired abilities to differentiate and also impaired functional capacities [15,16] that could be related with the decrease in HIF1α.

Our observations of increased glucose and glutamate metabolites in conditioned medium from obese ASCs are consistent with our previous findings of decreased glucose uptake in obese ASCs [15], reinforcing the notion that ASC metabolism is perturbed in obesity.

**Fig 7:**
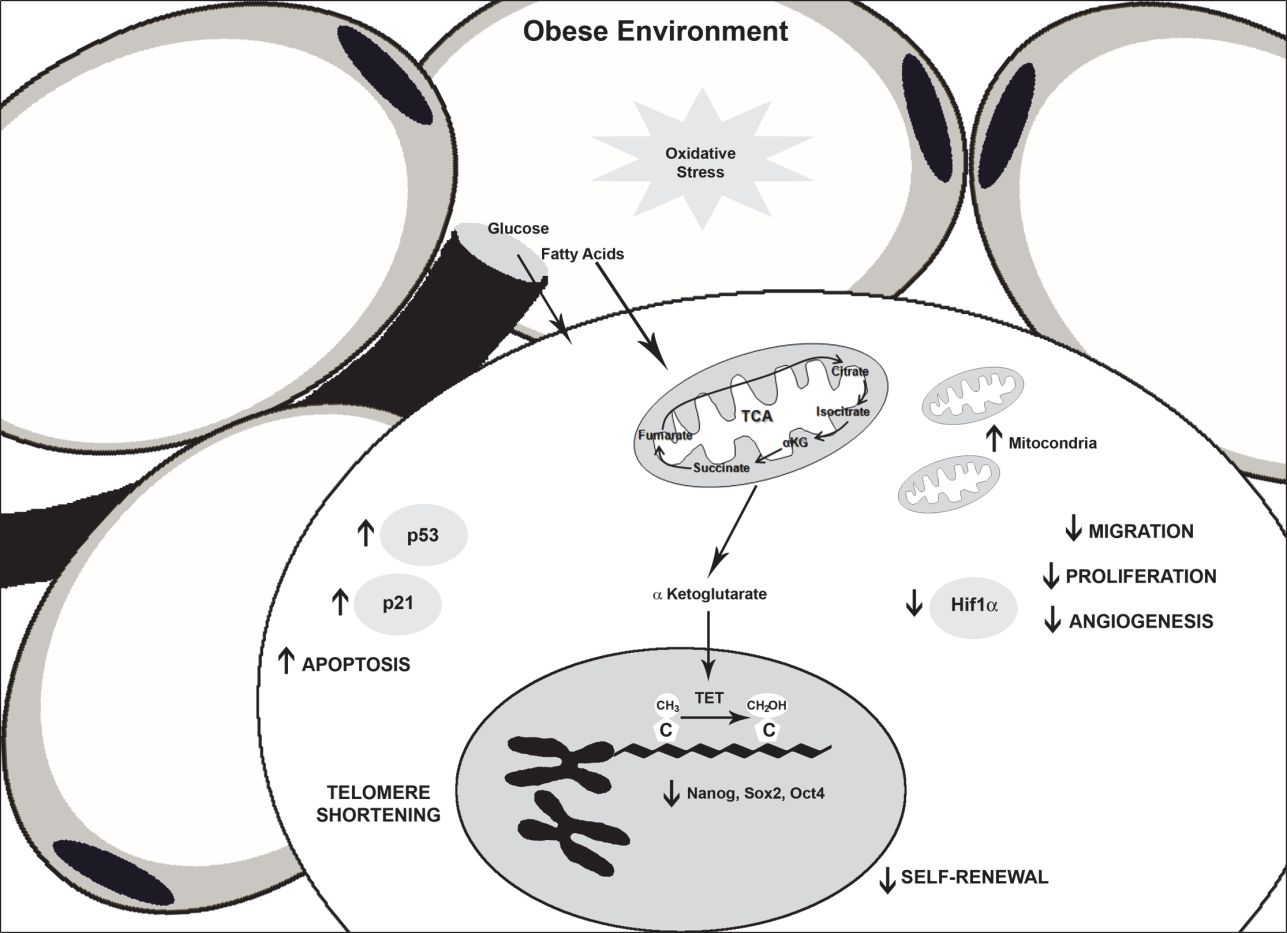
Altered general functions in obese ASCs. Marked dysfunction in stem cells from obese environments.

Our findings reported here together with our previous data allow us to propose the following model (Fig 7). An obese environment produces oxidative stress [56] that disturbs the ASC reservoir. Under obese conditions, there is an increase in the total mitochondrial mass of obese ASCs and these mitochondria have impaired respiration capacities in the presence of glucose but are better disposed to utilize fatty acids as a carbon source. Moreover, this increase of oxidative stress provokes telomere shorting. Additionally, there are changes in hydroxymethylation that are associated with a decrease in pluripotency gene expression and a decrease in self-renewal capacity. A reduction in *Hif1α* expression in obese ASCs could be related with the decrease in proliferation, migration and differentiation capacities. Expression of p21 and p53 were increased in obese ASCs, promoting cell apoptosis, and an absence of p16 and β-galactosidase staining suggested non-senescence processes. Finally, glucose levels detected by metabolite analysis in the extracellular medium corroborate the insulin resistant phenotype of obese ASCs and are consistent with reduced glucose uptake. Therefore, ASCs derived from obese subjects present a generalized metabolic dysfunction.

In conclusion, the present study demonstrates that ASCs taken from an obese environment are inferior in a variety of capacities. Clearly, these differences should be considered when obtaining mesenchymal stems cells from obese patients for cellular therapies.

## Supporting Information

S1 Fig OCR and ECAR profiles illustrated to define measurements in this study. (TIF)

S2 Fig Proliferation rate of ASCs. Growth rate (PDT^-1^) calculated using the population doubling time values obtained from proliferation curves in murine and human (A), at passage 10 and 20 and in nonobese and obese ASCs. Data represent mean±SEM from three replicates. Cell proliferation was measured by BrdU incorporation assay (B) in murine and human at passage 10 and 20 and in nonobese and obese ASCs after 16 h. Data represent mean±SEM from three replicates. * p <0.5, **p<0.01.
(TIF)

S3 Fig Extracellular lactic acid in ASCs. Measurements of lactate levels in ASCs from murine and human, showing quantity (nmol) per mg of protein. Data represent mean±SEM from three replicates. **p<0.01, ***p<0.001.
(TIF)
