# Supplementary figures and images for: Altered Metabolic and Stemness Capacity of Adipose Tissue-Derived Stem Cells from Obese Mouse and Human
Source: PLoS One. 2015 Apr 13;10(4):e0123397. doi: 10.1371/journal.pone.0123397 (PMC4395137; doi:10.1371/journal.pone.0123397)

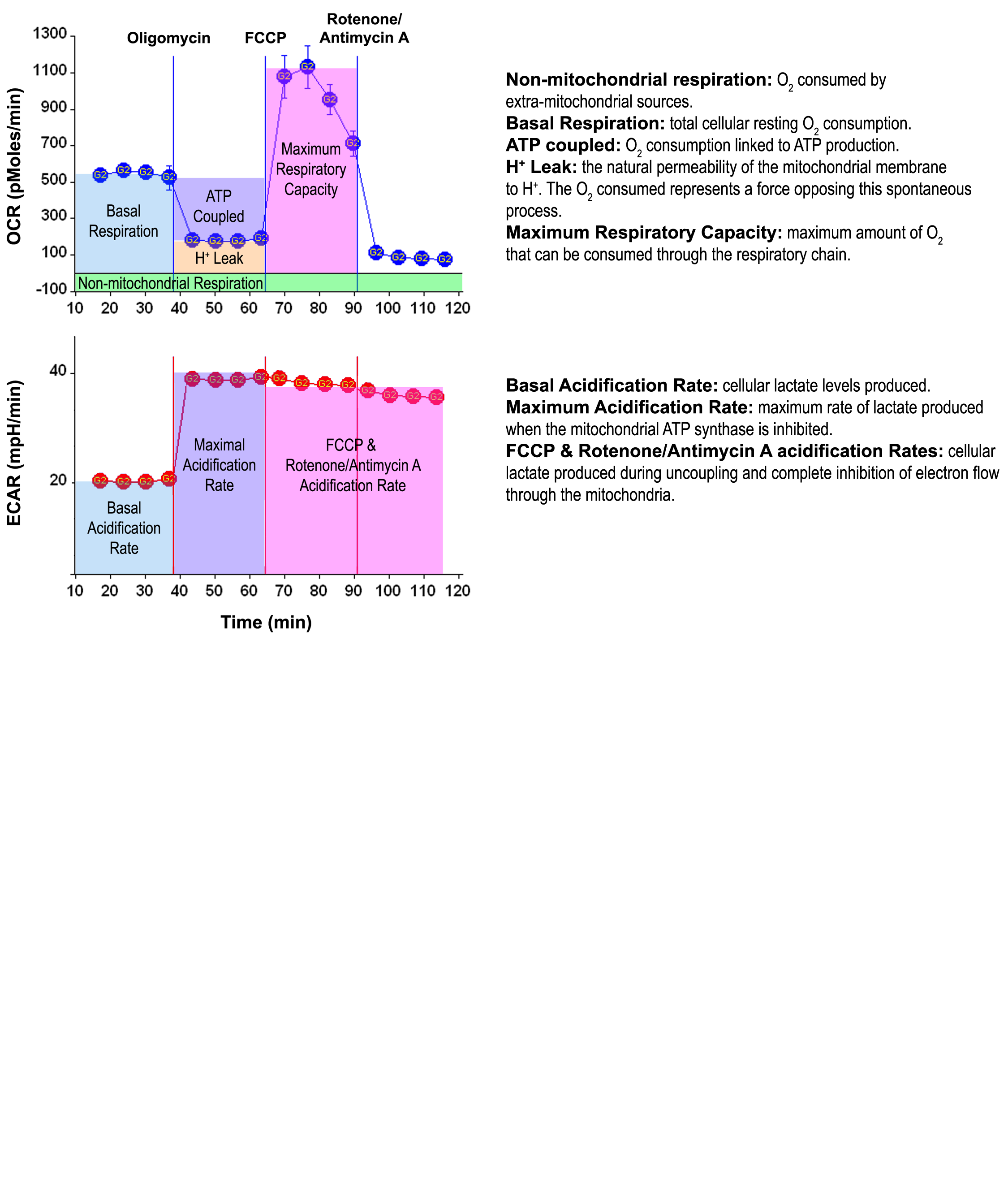

Supplement: S1 Fig — (TIF) [file pone.0123397.s001.tif]

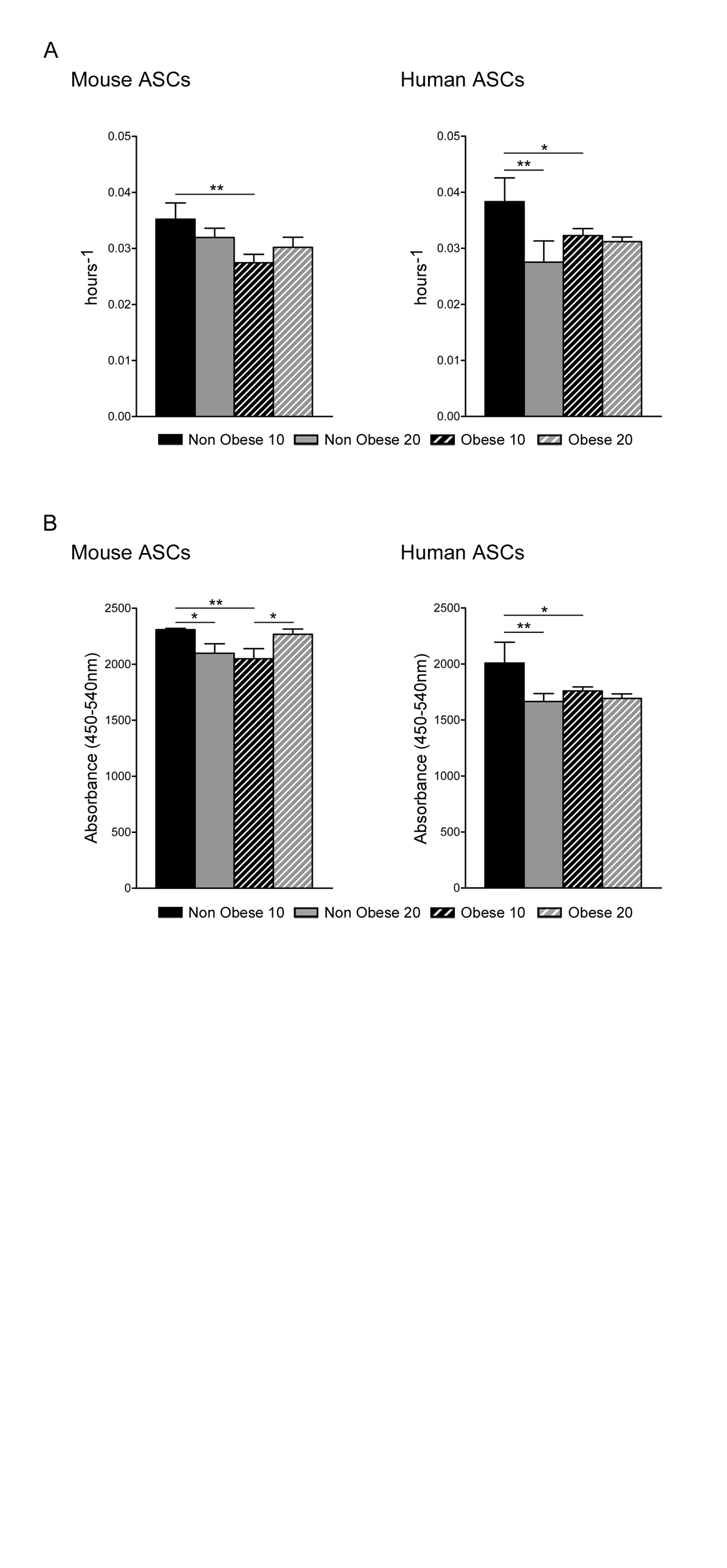

Supplement: S2 Fig — Growth rate (PDT-1) calculated using the population doubling time values obtained from proliferation curves in murine and human (A), at passage 10 and 20 and in nonobese and obese ASCs. Data represent mean±SEM from three replicates. Cell proliferation was measured by BrdU incorporation assay (B) in murine and human at passage 10 and 20 and in nonobese and obese ASCs after 16 h. Data represent mean±SEM from three replicates. * p <0.5, **p<0.01. (TIF) [file pone.0123397.s002.tif]

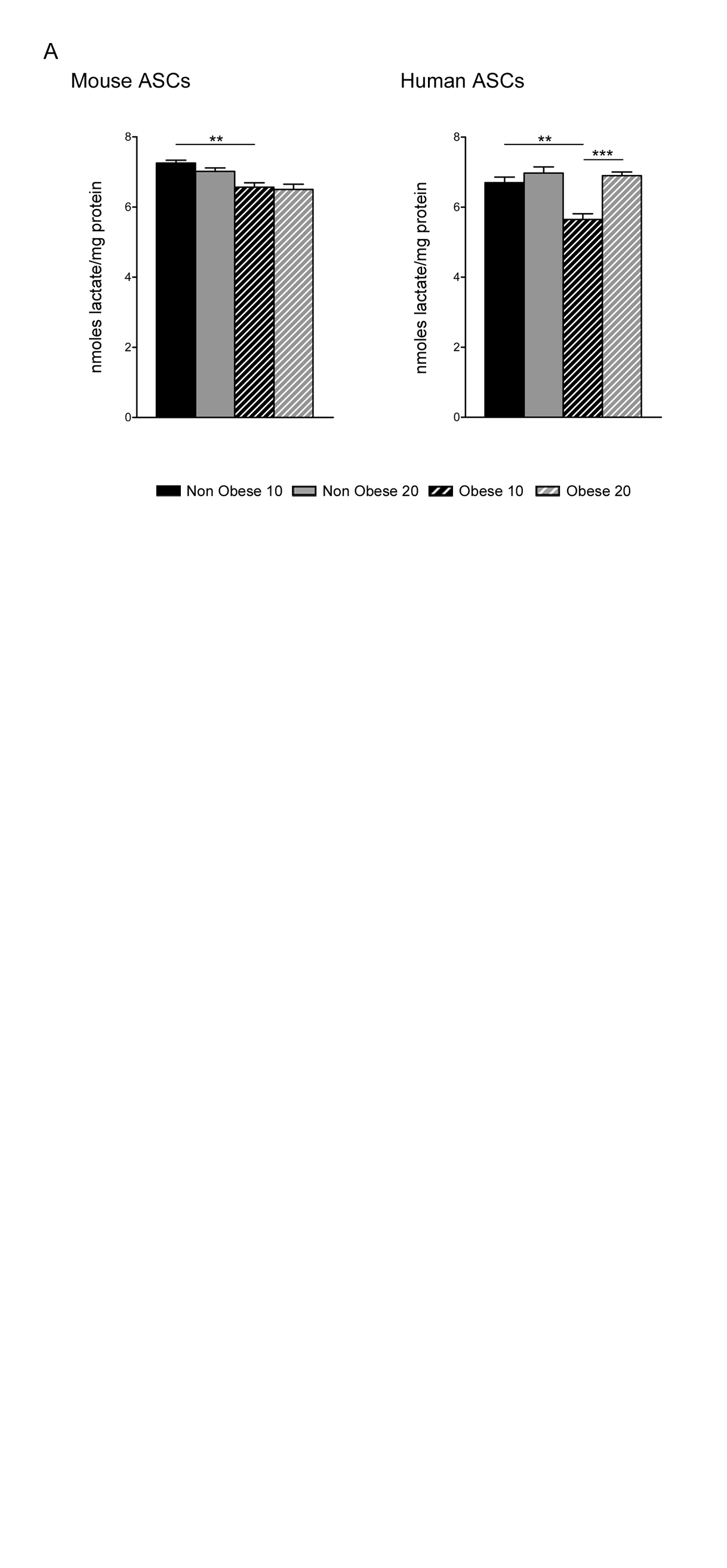

Supplement: S3 Fig — Measurements of lactate levels in ASCs from murine and human, showing quantity (nmol) per mg of protein. Data represent mean±SEM from three replicates. **p<0.01, ***p<0.001. (TIF) [file pone.0123397.s003.tif]
